# Human papillomavirus in women infected with human immunodeficiency virus: association with viral load and lymphocyte count

**DOI:** 10.1590/S1678-9946202466036

**Published:** 2024-06-07

**Authors:** Ana Cléa Cutrim Diniz de Morais, Alice de Sá Ferreira, Carla Déa Trindade Barbosa, Maria Fernanda Bezerra Lima, Karina Donato Fook, Mônika Machado de Carvalho, Alessandra Costa de Sales Muniz, Deborah Rocha de Araújo, Pablo de Matos Monteiro, Maria José Abigail Mendes Araújo, Sally Cristina Moutinho Monteiro, Fernanda Ferreira Lopes

**Affiliations:** 1 Universidade Federal do Maranhão, Programa de Pós-Graduação em Saúde do Adulto, São Luís, Maranhão, Brazil; 2 Universidade Federal do Maranhão, Hospital Universitário, Laboratório de Análises Clinicas e Histocompatibilidade, São Luís, Maranhão, Brazil

**Keywords:** Human papillomavirus, Human immunodeficiency virus, Coinfection

## Abstract

Women living with human immunodeficiency virus are at an increased risk of developing cancers related to human papillomavirus (HPV). Thus, it is important to combine clinical assessments, serological screening, and HPV data for planning prevention policies. This study aimed to identify HPV and its specific types in the cervical, anal, and oral mucosa of HIV-seropositive women, associating it with viral load and lymphocyte count. Sociodemographic characteristics, health data (CD4+ and CD8+ T cell counts and viral load), and biological samples (cervical, anal, and oral) were collected from 86 HIV-positive women undergoing antiretroviral therapy. Data were classified according to the presence or absence of HPV-DNA, HPV-DNA presence at one or more anatomic sites, and level of oncogenic risk, considering low- and high-risk oncogenic HPV-DNA groups. The presence of HPV in the cervicovaginal site was 65.9%, 63.8% in anal canal, and 4.2% in oral mucosa. A viral load ≥75 HIV copies/mL was associated with the presence of HPV-DNA. There was an association between viral load and the low-risk HPV or high-risk HPV groups. We found a high prevalence of HPV infection in HIV-seropositive women, particularly in the cervical and anal mucosa, with viral load ≥75 HIV copies/mL being associated with HPV-DNA presence.

## INTRODUCTION

Human papillomavirus (HPV) is a major risk factor for the development of preneoplastic and neoplastic intraepithelial lesions and cervical cancer^1^. HIV-positive women have a 2.7 (1.8–4.0) times higher incidence rate of cervical cancer compared to the general population^2^. In addition, HIV-positive men and women are 1.5 to 3 times more likely to develop cervical cancer than oropharyngeal cancer^3,4^.

In a meta-analysis of women living with HIV, HPV prevalence was found to be 42.6%^5^ and, in a more recent study with HIV-seropositive women, the prevalence of HPV infection was 36%^7^. HPV 16 (8.1%) and HPV 18 (3.7%) were the most prevalent in HIV-positive women^7^. A recent meta-analysis involving 37 studies and 8,436 HIV-positive women revealed a combined HPV prevalence of 62%, with a wide variety of high-risk HPV genotypes, with low TCD4+ counts being the factor most associated with high-risk HPV^8^.

The use of antiretroviral therapy reduces opportunistic infections and certain neoplasms related to acquired immunodeficiency syndrome (AIDS)^9^, but there has been an increase in virus-associated cancers, such as HPV-related anal and cervical cancer^10^. Moreover, the frequency of CD4+ T cells decreases in HIV+ women with coexisting HPV infection, contributing to increased HPV infection and persistence rates in HIV+ women^11^.

A major obstacle to planning prevention policies has been the lack of data, which is partly because serological screening does not include HPV screening, or because a lack of integration of clinical data from patients sometimes occur. In addition, HPV genotyping is an important test that can contribute to the prevention of cervical and anal cancers and is essential for a better understanding of high-risk HPV types and the introduction of an effective immunization program.

Thus, this study aimed to identify the presence of human papillomavirus and its specific types via HPV genotyping in the cervical, anal, and oral mucosa of seropositive women for the human immunodeficiency virus, associating it with the viral load and lymphocyte count. Moreover, we aimed to identify the prevalence of HPV-DNA in cervical, anal, and oral mucosa, relating its presence and specific types with sociodemographic and laboratory data.

## MATERIALS AND METHODS

### Study type and location

This is a cross-sectional, quantitative study conducted from January 2020 to December 2021 at the Reference Centers for HIV treatment in Sao Luis city, Maranhao State, in Northeastern Brazil. This research was approved by the Research Ethics Committee of the University Hospital of the Federal University of Maranhao (CAAE 70989617.4.0000.5086). All participants signed an informed consent form.

### Study population

A total of 86 HIV-positive women were included in this study. Only women aged from 20 to 69 years and who were undergoing antiretroviral treatment were included. Exclusion criteria consisted of pregnancy, lactation, menstrual period, contraindications for the Pap smear (such as current use of vaginal eggs or vaginal douches in the last 24 hours before biological sample collection), immunocompromised women by a biological agent other than HIV (previously diagnosed), and hysterectomized women.

### Data collection and biological sample

A structured questionnaire was applied on sociodemographic data (age, age at first sexual intercourse, marital status, number of sexual partners, number of children) and information on drug treatment. Data on TCD4+, TCD8+ cell counts (cells/mm3), and viral load (copies/mL) were obtained from the electronic medical records of the specialized service. Clinical specimens were collected from endocervical, anal, and oral mucosa by inserting a swab. These samples were stored in Tris-EDTA buffer pH 7.4, kept in a freezer at −20 °C, and subjected to HPV testing at the Laboratory of Genomic and Histocompatibility Studies (LEGH) and Laboratory of Molecular Biology of the Graduate Program in Adult Health (PPGSAD) at the Federal University of Maranhao (UFMA).

### Sample processing

Nucleic acid extraction was performed from the swabs using the QIAamp DNA Mini Kit™ (Qiagen), following the manufacturer's manual. DNA-HPV detection was performed using DNA extracted and subjected to amplification by the Nested polymerase chain reaction (PCR) technique, using two sets of primers PGMY09/11 (amplifying sequences of 450 bp from the L1 region of the viral DNA) and GP+5 and GP+6 (amplifying sequences of 190bp)^12^, Moreover, the amplification was conducted using the Thermal Cycler PCR-VERITI Dx 96 well (Applied Biosystems, Thermo Scientific, California, USA).Known HPV-positive samples were used as positive reaction controls, whereas ultrapure water was used as negative control. The amplified products were observed by agarose gel electrophoresis (1.5%), stained with 0.1% DNA Gel Red intercalator, and visualized in an ultraviolet light transilluminator (BIO-RAD Laboratories, USA).

HPV-DNA-positive Nested PCR products were purified with the Genelute PCR Clean-up kit, following the manufacturer's protocol (Sigma-Aldrich, USA). The genotyping reactions were subjected to the Sanger sequencing technique, by automatic sequencer AB 3500 Genetic Analyzer equipped with 50 cm capillaries and POP7 polymer (Applied Biosystems). The DNA templates were purified with the ExoSAP-IT™ PCR Product Cleanup reagent (Applied Biosystems) and quantified on the Nanodrop 2000c instrument (Thermo Scientific).

### Statistical analysis

Data were categorized into two groups: HPV-DNA-negative (HPV-DNA-negative) HIV-seropositive women and HPV-DNA-positive (HPV-DNA-positive) HIV-seropositive women. HPV-DNA-positive participants were subdivided into HPV-DNA in one anatomical site and HPV-DNA in more than one anatomical site. After genotyping, a subdivision into low-risk HPV-DNA and high-risk HPV-DNA was performed. Thus, categorical variables were evaluated by the chi-square test or the G test, whereas numerical variables were subjected to the Shapiro-Wilk normality test, with a non-parametric distribution. Thus, the Mann-Whitney test was used to compare the two groups. A significant p-value <0.05 was considered. The tests were performed using the IBM SPSS version 24 program.

## RESULTS

The sample consisted of 86 HIV-seropositive women undergoing antiretroviral treatment, of which 47 (54.7%) were positive for the HPV-DNA test. The median age of the group of HPV-DNA-negative and HPV-DNA-positive women showed no statistical difference, as well as self-reported skin color, marital status, number of children, age at first sexual intercourse, use of hormonal contraceptive method, and the number of sexual partners (p>0.05), as depicted in Table 1.

**Table 1:** Sociodemographic characteristics of HIV-seropositive women with and without HPV-DNA co-infection, Sao Luis city, Maranhao State, Brazil, 2021-2022.

Characteristic		HPV-DNA negative Median (95%CI)	HPV-DNA positive Median (95%CI)	*p*-value
Age		46.50 (34.5-59.2)	43.0 (34-52.5)	0.7
Characteristic		**HPV-DNA negative 39 (45.3%)**	**HPV-DNA positive 47 (54.7%)**	***p*-value**
Ethnicity				
	Black	23 (59)	27 (57.4)	0.9
	White	10 (25.6)	12 (25.5)	
	Indigenous	6 (15.4)	8 (17)	
Marital status				
	with partner	24 (61.5)	19 (41.3)	
	no partner	15 (38.5)	27 (58.7)	0.06
Number of sexual partners				
	>2	5 (12.8)	12 (25.5)	
	≤2	34 (87.2)	35 (74.5)	0.1
Children				
	Yes	35 (89.7)	43 (91.5)	0.7
	No	4 (10.3)	4 (8.5)	
Age at first sexual intercourse				
	<12 years	5 (12.8)	5 (10.6)	0.7
	≥12 years	34 (87.2)	42 (89.4)	
Hormonal contraceptive				
	Yes	1 (2.6)	1 (2.1)	1.0
	No	38 (97.4)	46 (97.9)	

Mann-Whitney, G-test, Chi-square, and Fisher's exact tests; α = 0.05.

When stratified by different anatomical sites, HPV-DNA presence in the cervicovaginal site was 65.9%, 63.8% in the anal canal, and 4.2% in the oral mucosa. In total, 29.8% were HPV-DNA positive in cervicovaginal and anal canals at the same time and 2.12% were positive in the cervicovaginal, anal, and oral canals, simultaneously. In the assessment of viral load and lymphocyte count, it was found that the viral load <75 HIV copies/mL was predominant in both groups, being 90% in the HPV-DNA-negative group and 78.3% in the HPV-DNA-positive group, with statistical significance (p<0.05) in the comparison between groups (Table 2).

In the lymphocyte count, it was found that TCD4+ and TCD8+ lymphocytes showed a higher median value in the DNA-HPV-negative group, with statistical significance (p<0.05), in turn, the CD4+/CD8+ ratio did not show significance between HPV-DNA negative versus HPV-DNA positive (p>0.05), as shown in Table 2.

**Table 2:** HIV viral load count and TCD4+ and TCD8+ lymphocyte counts of HIV-seropositive women with and without HPV-DNA co-infection, Sao Luis city, Maranhao State, Brazil, 2021-2022.

Factor		HPV-DNA negative 30 (%)	HPV-DNA positive 46 (%)	*p*-value
Viral charge				
	< 75 copies/mL	27 (90%)	36 (78.3%)	0.005
	≥75 copies/mL	3 (10%)	10 (21.7%)	
**Factor**		**HPV-DNA negative Median (95%CI)**	**HPV-DNA positive Median (95%CI)**	***p*-value**
CD4 cells/mm^3^		735.0 (601.75-888)	591.0 (377.5-745.7)	0.02
CD8 cells/mm^3^		812.0 (727-910)	676.0 (414-895)	0.02
CD4/CD8		0.88 (0.65-1.26)	0.84 (0.51-1.16)	0.6

Mann-Whitney, G-test, Chi-square, and Fisher's exact tests; α = 0.05

Table 3 shows that a viral load <75 HIV copies/mL was predominant in both groups (84% HPV-DNA in one anatomical site and 81.8% HPV-DNA in more than one anatomical site), without statistical significance (p>0.05). The TCD4+, TCD8+ count, and the CD4+/CD8+ ratio also did not show statistical significance between the DNA-HPV groups at one or more biological sites.

**Table 3:** HIV viral load count and TCD4+ and TCD8+ lymphocyte counts of HIV-seropositive women with HPV-DNA co-infection, by anatomical site, Sao Luis city, Maranhao State, Brazil, 2021-2022.

Factor		Positive HPV-DNA in one anatomical site (25)	Positive HPV-DNA in more than one anatomical site (11)	*p*-value
Viral charge				
	< 75 copies/mL	21 (84%)	9 (81.8%)	0.8
	≥75 copies/mL	4 (16%)	2 (18.2%)	
**Factor**		**Positive HPV-DNA at one anatomical site Median (95%CI) (N=34)**	**Positive HPV-DNA in more than one anatomical site Median (95%CI) (N=13)**	***p*-value**
TCD4 cells/mm^3^		652.5 (415-844)	692.0 (283-1091)	0.4
TCD8 cells/mm^3^		833.0 (461-1286)	895.0 (615-1085)	0.09
CD4/CD8		0.87 (0.49-1.78)	1.71 (0.89-18.00)	0.1

Mann-Whitney, G-test, Chi-square, and Fisher's exact tests; α = 0.05.

Viral load < 75 HIV copies/mL in the high-risk HPV-DNA group was present in 94.7% of the participants, demonstrating statistical significance (p<0.05) with the low-risk HPV-DNA group. Regarding the count of TCD4+, TCD8+ lymphocytes, and the CD4+/CD8+ ratio, no statistical differences were found (p>0.05) between the high-risk and low-risk HPV-DNA groups (Table 4).

**Table 4:** HIV viral load count and TCD4+ and TCD8+ lymphocyte counts of HIV-seropositive women with HPV-DNA co-infection, by type of oncogenic risk, Sao Luis city, Maranhao State, Brazil, 2021–2022.

Factor		Positive HPV-DNA in one anatomical site (25)	Positive HPV-DNA in more than one anatomical site (11)	*p*-value
Viral charge				
	< 75 copies/mL	12 (70.6%)	18 (94.7%)	0.04
	≥75 copies/mL	5 (29.4%)	1 (5.3%)	
**Factor**		**HIV + HPV low risk**	**HIV + high risk HPV**	***p*-value**
TCD4 cells/mm^3^		627 (474–853)	576 (151–775)	0.2
TCD8 cells/mm^3^		715 (439–744)	615 (388–914)	0.9
Relationship CD4/CD8		0.81 (0.28–1.10)	1.0 (1.0–1.0)	0.09

Mann-Whitney, G-test, Chi-square, and Fisher's exact tests; α = 0.05

## DISCUSSION

In this study, more than half of the participants (54.7%) were HPV-DNA-positive, similar to a previous study with HPV genotyping data in 213 HIV-seropositive women from Mbeya (Tanzania), of which 57% had HPV coinfection^11^. Conversely, varying prevalence of HPV have been reported among HIV-seropositive women, ranging from 31.1% to 63%^13-15^. Nonetheless, in a multicenter study with 802 HIV-seropositive women, 28.4% had HPV infection^7^, whereas a systematic review and a meta-analysis study reported HPV prevalence of 63% and 42,6% among HIV-seropositive women^7,15^. There are several explanations for these varying results on the frequency of HPV in HIV-positive women, including differences in sample size, population characteristics^15^, and methods used for HPV detection and genotyping^8^.

The data from this study showed a higher prevalence of cervical HPV infection compared to the oral and anal sites, and HPV-DNA was found in two or more anatomical sites. However, another study^16^ found a rate of oral HPV infection of 14.8% in HIV-seropositive women, higher than that of seronegative women (9.4%), with 41% of seropositive patients presenting HPV at the cervical and oral sites. We highlight that multiple HPV infections can be detected in the same individual across different anatomical areas^17^.

In this study, we found that the viral load of patients with HPV/HIV coinfection was predominantly less than 75 copies/mL. Specifically, 10% of the DNA-HPV-negative group and 21.7% of the HPV-DNA-positive group had a viral load ≥ 75 copies/mL, with statistical significance (p<0.05) between groups. This result is relevant since literature data suggest an association between the HIV viral load and the occurrence of cervical lesions, demonstrating that the viral load can significantly increase the risk of developing cervical lesions^18^. Thus, elevated viral load promotes persistent HPV infection and allows the recurrence of cervical lesion^19^.

In this study, as all participants were undergoing antiretroviral treatment and being followed up by healthcare professionals at specialized centers, the median CD4+ T cell count was higher than the standard for starting antiretroviral therapy (ART), which ranges from <350 to <500 cells per μL^20^. On the other hand, a significant (p<0.05) moderate decline in the median frequency of CD4+ T cells was observed in HIV-seropositive women with HPV coinfection compared to those without coinfection, as detected in another study^11^.

Moreover, TCD4+ count <100 cells/µL was associated with the presence of cervical HPV infection in HIV-seropositive women^20^, suggesting that a low TCD4+ count can be considered a risk factor for high-risk HPV infection, such as type 16^21^. HIV infection induces the systemic loss of CD4+ T cells, increasing the chances of developing opportunistic infections such as HPV^11^. Simultaneously, TCD4+ count greater than 500/μL has been indicated as a protective factor against the development of cervical lesions^22^.

HPV-negative participants and low-risk HPV carriers had higher CD8+ T cell counts. This result can be explained by the correlation between the CD8+ T cell and the initial decline in viral replication since HIV can show disordered replication without the presence of CD8+ T cells^23^.

In our study, CD8+ T cells were also associated with HPV infection, as women with HIV and HPV co-infection had lower CD8+ T cell counts. Our data corroborate another study^24^ that found a relationship between the reduction of CD8+ T cells in HIV-seropositive women and cervical lesions. CD8+ T cells show a high cytotoxic capacity and are associated with the control of viral replication and slow progression of HIV; their depletion may contribute to a greater probability of HPV infection as well as to the persistence of infection^25^.

The CD4+/CD8+ ratio in HIV-seropositive women is reflected in the cervical mucosa. The gradual decrease of CD4+ T cells and the decline in cell-mediated immunity among HIV-seropositive individuals may compromise the response to HPV infection and potentially act in accelerating the neoplastic process^26^.

We highlight that this study presents limitations such as the sample size. Notably, a strong point of the study is that the same participants were investigated for the presence of HPV in three different anatomical sites (cervical, anal, and oral) by nested-PCR assays. However, real-time PCR was not employed to quantify HPV viral load in positive samples. This study highlights the importance of constant monitoring of these patients, despite undergoing antiretroviral treatment, as we found a higher prevalence of HPV among HIV-seropositive women with higher viral load and lower CD4+ and CD8+ T cell counts.

## CONCLUSION

In conclusion, this study found that the presence of HPV is common among women who are seropositive for HIV. This confirms that HIV-seropositive women have a higher risk of infection and persistence of the human papillomavirus, with association with CD4+ and CD8+ cell counts and higher viral loads.
